# Poly-(ADP-ribose)-polymerase inhibitors as radiosensitizers: a systematic review of pre-clinical and clinical human studies

**DOI:** 10.18632/oncotarget.19079

**Published:** 2017-07-07

**Authors:** Paul Lesueur, François Chevalier, Jean-Baptiste Austry, Waisse Waissi, Hélène Burckel, Georges Noël, Jean-Louis Habrand, Yannick Saintigny, Florence Joly

**Affiliations:** ^1^ Laboratoire d’Accueil et de Recherche avec les Ions Accélérés, CEA, CIMAP-GANIL, 14000 Caen, France; ^2^ Centre Francois Baclesse Centre de Lutte Contre le Cancer, Radiotherapy Unit, 14000 Caen, France; ^3^ EA 3430, Laboratoire de Radiobiologie, Centre Paul Strauss, 67000 Strasbourg, France; ^4^ Centre Francois Baclesse Centre de Lutte Contre le Cancer, Clinical Research Unit, 14000 Caen, France

**Keywords:** radiosensitization, poly(ADP-ribose)-polymerase inhibitors, radiotherapy, radiobiology

## Abstract

**Background:**

Poly-(ADP-Ribose)-Polymerase (PARP) inhibitors are becoming important actors of anti-neoplasic agents landscape, with recent but narrow FDA's approvals for ovarian BRCA mutated cancers and prostatic cancer. Nevertheless, PARP inhibitors are also promising drugs for combined treatments particularly with radiotherapy. More than seven PARP inhibitors have been currently developed. Central Role of PARP in DNA repair, makes consider PARP inhibitor as potential radiosensitizers, especially for tumors with DNA repair defects, such as BRCA mutation, because of synthetic lethality. Furthermore the replication-dependent activity of PARP inhibitor helps to maintain the differential effect between tumoral and healthy tissues. Inhibition of chromatin remodeling, G2/M arrest, vasodilatory effect induced by PARP inhibitor, also participate to their radio-sensitization effect.

**Materials and Methods:**

Here, after highlighting mechanisms of PARP inhibitors radiosensitization we methodically searched PubMed, Google Scholar, Cochrane Databases and meeting proceedings for human pre-clinical and clinical studies that evaluated PARP inhibitor radiosensitizing effect. Enhancement ratio, when available, was systematically reported.

**Results:**

Sixty four studies finally met our selection criteria and were included in the analysis. Only three pre-clinical studies didn't find any radiosensitizing effect. Median enhancement ratio vary from 1,3 for prostate tumors to 1,5 for lung cancers. Nine phase I or II trials assessed safety data.

**Conclusion:**

PARP inhibitors are promising radiosensitizers, but need more clinical investigation. The next ten years will be determining for judging their real potential.

## INTRODUCTION

Poly-(adenosine diphosphate-ribose)-polymerase (PARP) is a family of enzymes involved in a wide number of cellular processes, including DNA replication, transcription, repair and cell death. PARP proteins have been studied for decades for notably their roles in DNA repair. PARP1 is the most abundant and active enzyme of the PARP family, but roles of other members including PARP2 and PARP3 in DNA damage responses is emerging. In fact, they play an important role by detecting the presence of damaged DNA and then by activating signalization pathways that promote appropriate cellular responses. PARP is involved in base excision repair (BER) by allowing the recruitment and activation of BER actors and consequently make easier the DNA single strand break (SSB) reparation. Further studies have shown that PARP-1 and PARP-2 were also implicated in DNA breaks repair by Non-Homologous End Joining (NHEJ) and Homologous Recombination (HR).

Increased PARP activity has been observed in numerous cancers, and has been sounded to be one possible mechanism of resistance to cell-death by DNA-damaging therapeutics. Then progressively, PARP proteins became a very interesting target for oncologic treatments, and first PARP inhibitors (PARPi) were designed at the end of the eighties. Indeed, 2005 year has been a real turning point for the development of PARPi as two high-value publications showed that dysfunction of homologous recombination such as in BRCA1 and BRCA2 mutated cells triggered a high sensitization to PARPi [1, 2]. PARPi leads to the persistence of DNA lesions normally repaired by homologous recombination. Then, such accumulation of non-repaired DNA lesions leads to cell death. Synthetic lethality was then confirmed as a serious avenue of therapeutic development. PARP inhibitors have been evaluated in clinical trials either as single agents or in combination with DNA damaging agents. Olaparib, the most developed PARPi, has been approved by the FDA for treatment of Ovarian and Prostate BRCA mutated cancers. Many others molecules are being developed such as rucaparib, talazoparib, niraparib, veliparib, or simmiparib.

**Figure 1:**
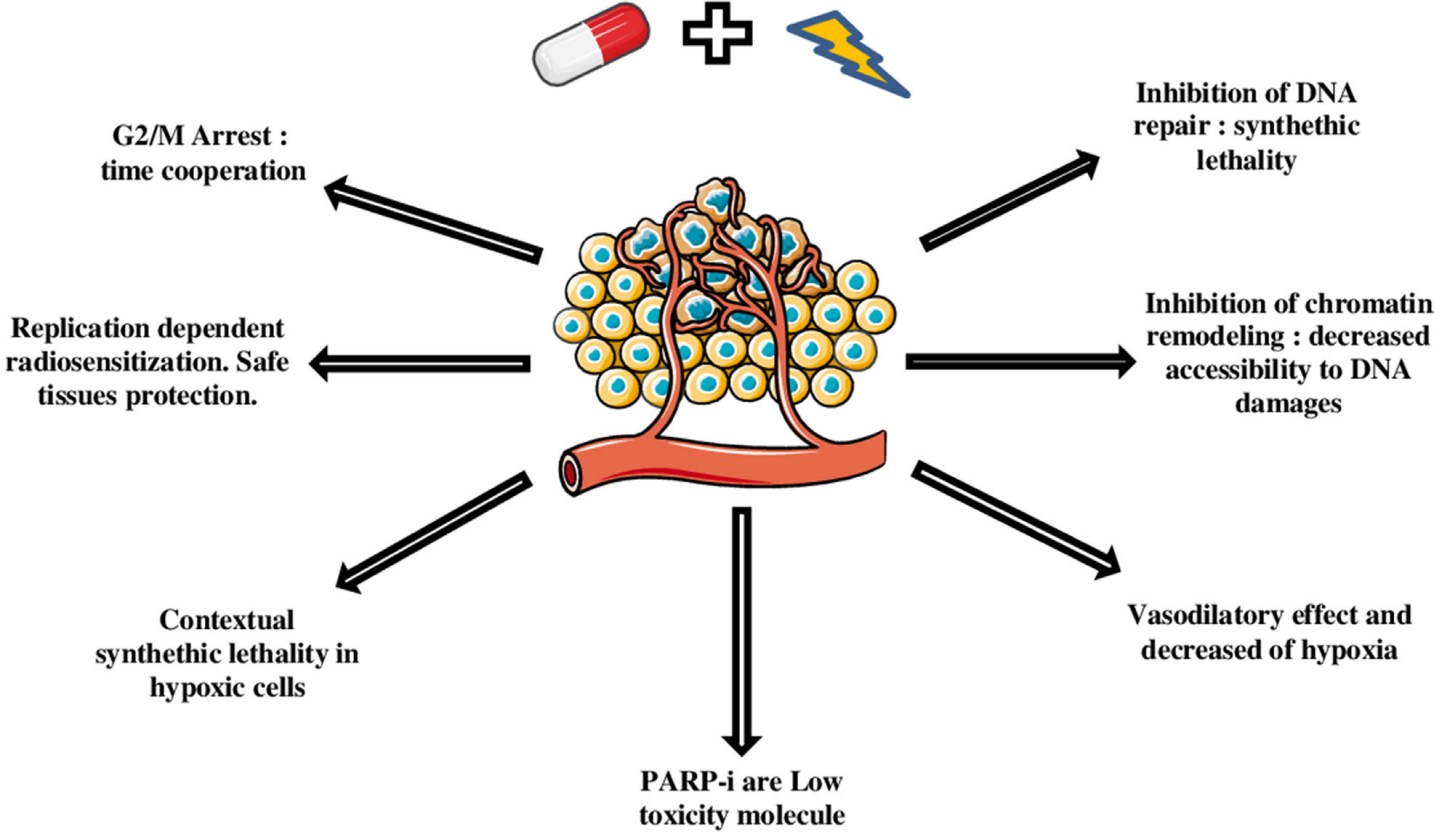
Mechanisms and advantages of PARPi radiosensitization

Classically, radiosensitivity is described as function of the tumor intrinsic radiosensitivity, the tumor repair capacity, the reoxygenation process, the cell cycle redistribution, and the tumoral tissue repopulation. More recently, with development of stereotactic radiotherapy, two additional characteristics appeared: the tumor immunity and the vascular endothelial damage process [3]. So, a radiosensitizer has to impact one or more of these processes without worsening the treatment toxicity. The combination of ionizing radiation with radio-enhancing agents represents an opportunity to increase the efficacy of radiotherapy as a treatment modality, and at the same time minimize toxic side effects and potential damages to healthy surrounding tissues. PARPi have many qualities required for radio sensitizing effects (Figure 1) and since the last ten years, they have become interesting molecules as radiosensitizers. There is evidence that the absence of PARP-1 and -2, which are both activated by DNA damage and facilitate DNA repair, produces an hypersensitivity to ionizing radiation. Therefore, the inhibition of PARP-mediated DNA damage repair can help to sensitize cells to radiation by prolonging strand breaks and by leading to a cell-death signaling pathway. This effect could be enhanced in cancers harboring defects in homologous recombination and by synthetic lethality mechanism. Nevertheless modulating DNA damage repair is not the only way of the radiosensistizing effect of PARPi.

The scope of this systematic review is to make a state of the art about advancements of PARPi use as radiosensitizer in human tumor cells and to provide food for clinician's thought who would wish to study radiosensitization methods. Each organs group will be treated separately. Before results of the systematic review, principal mechanisms of PARPI radiosensitization will be reminded.

### Mechanisms of radiosensitization of PARP inhibitors

### Inhibition of DNA repair

PARPs are involved in several DNA repair mechanisms such as: Base Excision repair (BER), homologous recombination (HR), conventional (c-NHEJ) or alternative non-homologous end joining repair (alt-NHEJ) [4–6].

PARP is one of the first actors of DNA single strand break (SSB) repair because of its role in BER. PARP is a part of the BER complex, described as a sensor of SSB. PARP-1 and 2 are the two most important PARP family enzymes implicated in BER. In fact, PARP detects SSB and leads to PARylation, meaning accumulation of poly-ADP-ribose (PAR) chain around the SSB [7, 8]. The local PARylation allows the recruitment of XRCC1 for break stabilization, DNA Polymerase β for the complementary base synthesis, and DNA ligase III for final ligation [9]. Auto-PARylation of PARP releases PARP from the SSB site. In case of PARP inhibition, PARP stays set on SSB site, BER machinery is not recruited, and SSB persists.

Associated with PARPi, radiation therapy would induce DNA damages, such as SSB or DSB which couldn't be repaired, and driving to DNA replication fork collapse. SSB is then converted in potentially lethal double strand break (DSB). PARPi are also trapped to DNA and induce “mechanical” replication fork collapse and consequently DSB [10]. It explains why PARP inhibition is more effective than PARP suppression [11]. That's the first and main radio sensitizing effect of PARPi. If homologous recombination deficient cells are considered, such as BRCA mutated or BRCAness ones [12], the effect is more potent: it is an example of synthetic lethality mechanism.

HR is an error free DNA repair mechanism in which PARP1-2 play again a sensor role, by making easier the recruitment of MRE11, DNA resection, and replication restart [10, 13]. Furthermore PARP-1 regulate RH, and can promote RH instead of error prone NHEJ. PARP inhibition prevents the use of efficient and reliable DNA repair system [5, 13].

There has been evidence, that an alternative end-joining (Alt-NHEJ) operates in the absence of the core components of classical NHEJ [14]. Alt-NHEJ is efficient, but highly mutagenic. PARP-1 is even known for his central role in alternative NHEJ [4]. PARPi suppress again this way of repair and lead to more DNA radiation damages.

As a conclusion the use of PARPi allow to target most of DNA repair system, so as to potentiate the radiotherapy effect by accumulating SSB and above all DSB.

### Replication-dependent radiosensitization

PARPi exercise their radiosensitizing effect, more specifically, during the cell cycle S phase [15]. For example with olaparib, in Dungey's glioblastoma cells study, radiosensitization was significantly more pronounced in S-phase (enhancement ratio at 50% survival: SER50 = 1.60) than in G1 (SER50 = 1.27) or G2 (SER50 = 1.33) enriched populations [16]. Moreover it is well known that tumor have a higher proliferating cell compartment in comparison to surround safe tissues. It means that PARPi may radiosensitize tumor tissue, while saving non tumoral tissue, which is one of the most important qualities of a radiosensitizing agent.

### Modulation of chromatin remodeling

PARP-1 dependent PARylation event directs the recruitment of helicase, such as ALC1 (amplified in liver cancer 1) to chromatin and nucleosomes [17, 18]. The helicase activity promotes the unwinding of DNA double helix and unblocks access to DNA of the machinery responsible for transcription, replication and DNA repair [17, 19]. After treatments by DNA damage agents just as irradiation, inhibition of PARP-1 could delay DNA double strand opening and DNA repair. It could be considered as another way of PARPi potential radiosensitization.

### Impact on microenvironnement and role of hypoxia

PARPi association with radiotherapy could help to bypass the hypoxia induced radioresistance. On the first hand, few PARPi, like rucaparib for example, have structural similarities with nicotinamide, a vaso-dilatory component. This specificity could enhance tumor growth delay after radiotherapy, by increasing tumor blood flow, enhancing drug penetration, and increasing oxygen concentrations to offset hypoxic cell radioresistance [20]. It could explain why sometimes PARP efficiency is higher *in vivo* than *in vitro* [20]. PARPi radiosensitize hypoxic tumor thanks to an oxygen effect. Ionizing radiation depends heavily on the presence of molecular oxygen to produce cytotoxic effect. The molecular oxygen O2 is absolutely necessary to chemically fix DNA free radicals produced by ionizing radiation [21]. In the absence of O2, DNA radicals are repaired by abstracting hydrogen from sulfhydryl (SH) group present in protein [21]. It has been reported that three times higher ionizing radiation dose is required to kill hypoxic cancer cells, compared to well-oxygenated cells, in order to achieve the equivalent level of cell kill [22, 23].

On the other hand, even without any improvement of the vasculature, PARPi exibit a radiosensitizing effect in hypoxic cells. In fact hypoxia generates a genetic instability by a mutator phenotype effect [24] linked to the decreased transcription of proteins involved in homologous recombination [25]. When PARPi and radiotherapy are combined in hypoxic conditions, we could observe contextual synthetic lethality. HR is altered by hypoxia and carries out an increased death ratio [26].

### G2/M arrest

With DNA repair, cell cycle regulation is perhaps the most important determinant of ionizing radiation sensitivity. A common cellular response to DNA-damaging agents is the activation of cell cycle checkpoints, leading to cell cycle arrest [27]. The concomitant radio-chemotherapy induces temporo-spatial cooperation. Spatial cooperation means that chemotherapy allows to cure overfield micro metastatic disease, whereas radiotherapy goal is to treat local invasion. Temporal cooperation means that chemotherapy synchronizes, and arrests cells in the radiosensitive phases of the cell cycle: G2 and M. In this context of temporal cooperation, chemotherapy could be considered as a radiosensitizer. PARPi could take part into the radiosensitization process in the same way as a result of the G2/M arrest induced, secondary to chromosomic aberrations generated by PARPi [1].

### Low toxicity molecule

Most used radiosensitizers, such as Cisplatin or Cetuximab, induce major systemic secondary effects, which could limit their use in clinical practice particularly for elderly patients such as: neuropathy, cytopenia, nephropathy, cutaneous toxicity. In phase II-III clinical trials studying PARPi monotherapy, toxicity remains manageable and consists most of the time of anemia, thrombocytopenia, neutropenia, asthenia and nauseas rarely upper than grade II [23–26].

This low toxicity lets suggest that PARPi use as radiosensitizer shouldn't worsen treatment safety.

### PARPi available or being developed

First PARPi were born at the beginning of the eighties and were derived from 3-aminobenzamide. Due to its lack of potency and specificity, 3-AB is not clinically useful. Therefore, a number of third-generation PARP inhibitors, some derived from the 3-AB structure, have been developed in recent years and tested in pre-clinical and clinical studies. Their development has been faster during the second half of 2000’s, corresponding to the discover of anti tumoral response in BRCA mutated cells by Bryant and Farmer [1, 2]. PARPi suppress activity of PARP catalytic domain explaining synthetic lethality in HR defective cells. Nevertheless, PARP inhibition, delays SSB repair to a greater extent than PARP depletion [11]. To explain these results, a PARP-1 trapping has been proposed based on the idea that PARP1 is trapped on DNA by PARP inhibitors, and PARP1-DNA complexes can interfere with DNA fork replication [32, 33].

Actually seven PARPi are being developped by pharmaceutical industry in clinical trials: Olaparib, Rucaparib, Niraparib, talazoparib, veliparib, CEP 9722, Simmiparib. They are all oral drugs. Among them only Rucaparib, Olaparib, Niraparib and Veliparib have been used as radiosensitizers. Others PARPi such as LT626, PJ34, GPI 21016, 3-Aminobenzamide or 4-amino-1,8-naphthalimide have been less employed, and only in pre-clinical studies (Table 1).

**Table 1:** PARP inhibitors and their use as radiosensitizers in pre-clinical and clinical research

Name	PARP Targeted	Development as radiosensitizer	EAM /FDA approved	Indication
Olaparib	PARP1-2	Phase I	No	None
Rucaparib	PARP1-2	Pre-Clinical	No	None
Veliparib	PARP1-2	Phase II	No	None
LT 626	PARP-1	Pre-clinical	No	None
PJ34	PARP1-2	Pre-clinical	No	None
GPI 21016	PARP1	Pre-Clinical	No	None
4-amino-1,8-naphthalimide	PARP1	Pre-Clinical	No	None
3-Aminobenzamide	PARP1	Pre-Clinical	No	None
Niraparib	PARP1-2	Pre-clinical	No	None

## MATERIALS AND METHODS

We have led our bibliographic research in accordance with PRISMA guidelines [34]. We looked for all clinical or pre-clinical studies related to the use of PARPi as radiosensistizers. We have requested PubMed, Cochrane and Google Scholar databases without any date limite and thus all the archives of meeting abstracts or posters of ASTRO, ESTRO, ESMO and ASCO congresses from 2010 to 2016. For PubMed Database, the key words research strategy was: “(((radiosensitization) OR radiotherapy)) AND (((((((niraparib) OR talazoparib) OR rucaparib) OR veliparib) OR olaparib)) OR “Poly(ADP-ribose) Polymerase Inhibitors” [Mesh])” We have selected all research papers published in English language until June 2017 7th. Studies dealing with animal cells were not included in the systematic review. Then we have excluded reviews, and articles which finally didn't treat about radiotherapy, such as, for example, use of radionuclides. After manuscripts reviewing and application of inclusion criteria, we have sorted the articles function of research stage: *in vitro*, *in vivo* or clinical studies. For each selected article we have extracted an enhancement ratio which refers to the enhancement effect of radiation due to the addition of PARPi. Enhancement ratio is classically a ratio between doses associated with surviving fractions of 10%, 37% or 50% with or without the PARPi. For example: SER37 = D37(no drug)/D37(PARPi). When enhancement ratio (ER) wasn't communicated as for *in vivo* studies, clinical studies or few *in vitro* studies, we have supplied a significant data such as survival, tumor growth delay. Then data are assembled and discussed by organ groups.

## RESULTS

**Figure 2:**
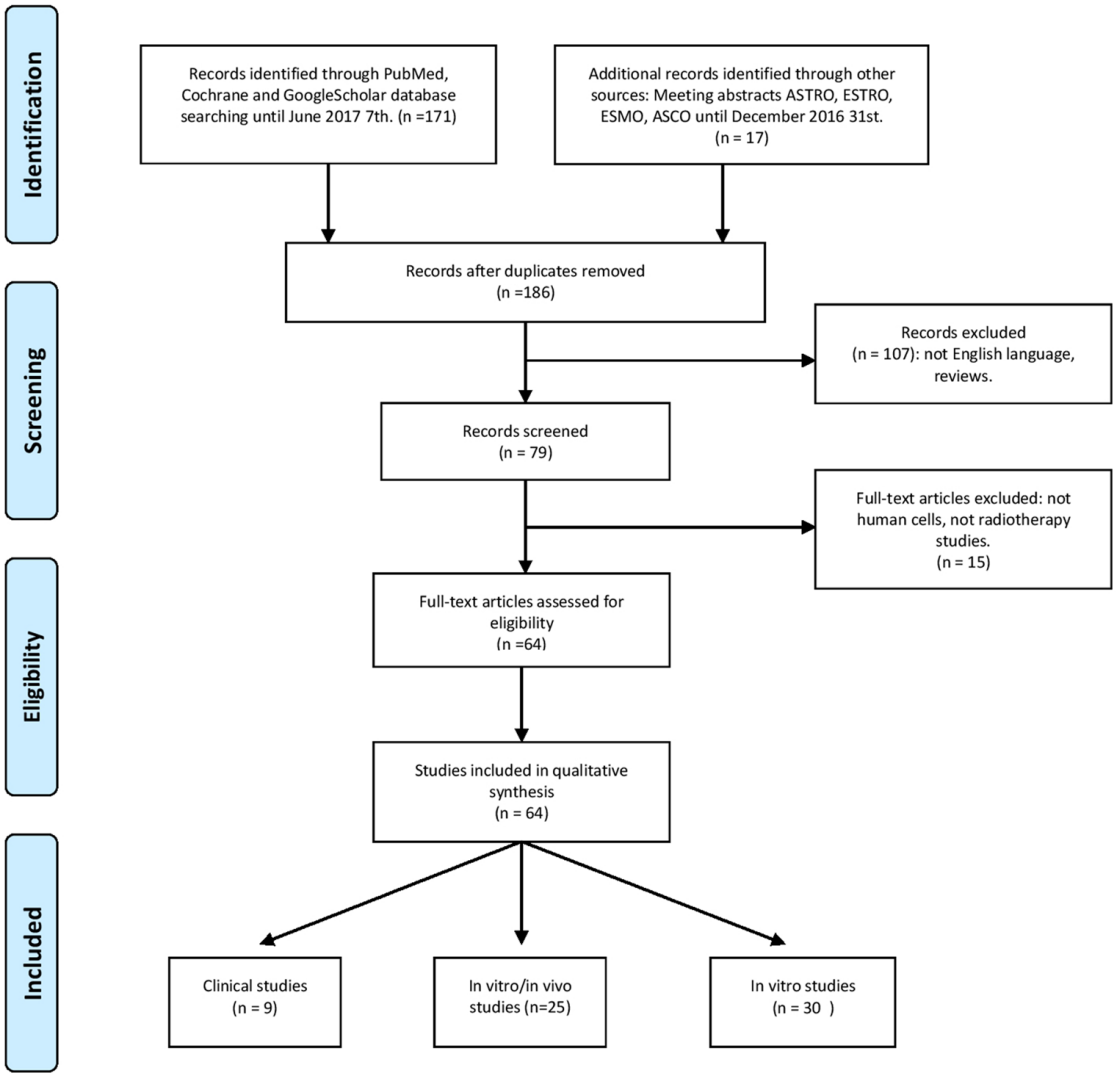
Flow chart of the systematic review following PRISMA guidelines

After applying our selection criteria, 60 studies have been included from Pubmed and Google scholar analysis. Four more clinical studies have been added after reviewing European, and American congress meeting abstracts, since 2010. Fifty five of the 64 selected studies are pre-clinical *in vitro* or *in vivo* studies. For 49% of pre-clinical studies, it has been possible to extract an enhancement ratio. For others studies, a significant result about the efficacy of the association has been given. Studies about brain or digestive tumors represent 52% of studies dealing with PARPi as radiosensitizer. Median enhancement ratio vary from 1,30 for prostate tumors to,5 for lung cancers. Until June 2017 7th, there was no data with Talazoparib, Simmaparib or CEP 9722 use with radiotherapy. It is worth noting that all studies meeting our review selection criterias, are presented in the tables (Figure 2).

### PARPi + radiotherapy for brain tumors

Glioblastoma is probably the tumor which could benefit he most from radiosensitizing drugs. Indeed, glioblastoma is considered as a radioresistant tumor with a high level of intra-field recurrence. Furthermore the target is surrounded of high risk complications healthy tissues. Contrary to glioblastoma cells, neurons don't seem to express PARP-1 [49] and are low replication cells: this aids to protect healthy tissues by radiosensitizing only tumor cells. Glioblastoma stem cells promote radioresistance and could be the source of tumor recurrence after radiation [50]. Results from Venere *et al*. studies highlight constitutive activation of PARP1 in glioblastoma stem cells that can be therapeutically exploited. They showed that PARPi plus radiation compromises the stem cell phenotype *in vivo* and inhibits glioblastoma stem cells enrichment [51]. Concurrent Temozolomide (TMZ) + Irradiation followed by adjuvant TMZ is standard-of-care for patients suffering from glioblastoma [52]. In that case PARPi –inh could be considered as a radio and chemosensitizing drugs [53, 54]. PARPi lead to glioblastoma cells radiosensitization with most of *in vitro*/*in vivo* studies which are promising with radiosensitizing enhancement ratio comprised between 1,05 and 1,93 (Table 2). Thanks to inhibition of BER, PARPi prevent repair of N7-guanine and N3-adenine methylation induced by TMZ, increase DNA strand breaks and is responsible of a TMZ chemosensitization [53, 54] even on glioblastoma stem cell [55]. Data concerning the role of PARPi as TMZ chemosensitizer depending on MGMT status stay debated [40, 56–58]even if some phase II-III clinical trials recruits only patients with methylated MGMT (NCT0215298). Moreover, PARPi could induce synthetic lethality in PTEN deleted glioblastoma (36% of Glioblastoma) suggesting that it might be logical to treat PTEN-deficient glioblastomas with PARP inhibitors in the future [59]. In summary, PARPi, in combination with radiation therapy induce a median enhancement ratio of 1,30.

**Table 2:** Studies concerning PARPi radiosensitization for brain tumors

Brain system					
Cell lines / tumor	Phase	PARP Inhibitor	ER	Comments	Ref
Glioma cell lines (T98G and U373-MG)	*In vitro*	PJ34 (3 μM)	Yes (not communicated)	T98G sensitized by PJ34 to a range of low doses of radiation. U373-MG cells showed no increase of radiosensitivity for low-dose range (1 Gy).	[35]
Glioma cell lines (T98G, U373-MG, U87-MG,UVW)	*In vitro*	Olaparib (1 μM)	1,08 to 1,38	Radiosensitization is replication dependent and greater for fractionated than for single-dose treatments	[16]
Glioma cell lines (M059J and M059K)	*In vitro*	4-amino-1,8-naphthalimide (30 μM)	No	No significant increase of radiosensistivity	[15]
Glioma cell line (U251)	*In vitro In vivo*	*In vitro* GPI-21016(3 μM).*In Vivo* GPI-21016 (40 mg/kg) +TMZ(3 mg/kg)	1,6	*In vivo*: Absolute growth delay 31d (1,53x)	[36]
Glioma cell lines (SF188 and KNS42)	*In vitro*	Olaparib (0–8 μM)	Yes (not communicated)	Regression of cell Proliferation	[37]
Glioma cell lines (T98G, LN18, U87 and U251)	*In vitro*	Veliparib (5 μM) + TMZ (5–10 μM)	1,13 to 1,37 (Veliparib). 1,25 to 1,44 (Veliparib+TMZ)	Also measured in one of the MGMT-unmethylated cell lines with a SER50 value of 1.30	[38]
Glioma cell lines (UVW/NAT)	*In vitro*	Rucaparib(1 μM) / Olaparib (1 μM)	Rucaparib 1,33 Olaparib 1,91	PARP-1 inhibition in combination with X-irradiation promoted G2/M arrest	[39]
Glioblastoma cell lines derived from patients tumors (MGMT unmethylated)	*In vitro In vivo*	Veliparib (10 μM) Veliparib (12,5 mg/kg 2x/d)	Yes (not communicated)	Significant increase of mouse survival (+ 10days).	[40]
Primary patient-derived glioblastoma cell lines	*In vitro*	Olaparib (not available)	1,93 for Cancer Stem cells 1,34 for bulk cells	No significant difference between bulk and stem cells.	[41]
Pediatric Glioma cell lines (SJG2, SF188, KNS42)	*In vitro In vivo*	Niraparib (1 μM) Niraparib (50 mg/kg)	Yes (not communicated)	Significant decrease of survival fraction. *In vivo* survival : 1,6x longer	[42]
DIPG cell lines (DIPGM36 DIPG58, and SU-DIPG-IV)	*In vitro*	Niraparib (1 μM)	Yes (not communicated)	Significant decrease of survival fraction.	[42]
Ependymoma cell line (Res196)	*In vitro*	Olaparib (0–8 μM)	Yes (not communicated)	Regression of cell Proliferation. Decrease of clonogenic survival	[37]
Medulloblastoma cell lines (D283-med, D556-med and UW228-2)	*In vitro*	Olaparib (0–8 μM)	Yes (not communicated)	Persistance of γH2AX foci up to 72 hours after radiation. Regression of cell proliferation	[37]
Neuroblastoma cell lines (SH-SY-5Y, Kelly, NB1691luc and Tet 21)	*In vivo In vitro*	Nirabarib (50 mg/kg)	Yes (not communicated)	In vivo Survival: ≈ 1,08 x longer	[43]
Neuroblastoma cell lines (SK-N-BE(2c))	*In vitro*	Rucaparib(1 μM) Olaparib (1 μM)	Rucaparib 1,05 Olaparib 1,09	PARP-1 inhibition in combination with X-irradiation promoted G2/M arrest	[39]
Neuroblastoma cell (HX142c)	*In vitro*	3 amino benzamide (6 mM)	1,18	No impact of dose rate.	[44]
Glioblastoma	Phase I	Veliparib (10 mg bid) + TMZ(75 mg/m2)	not applicable	Only safety results.	[45]
All histologies Brain metastases	Phase I	Veliparib (escalating doses of 10–300 mg bid) + Whole brain radiotherapy	not applicable	OS: 10 m Vs 3,5 m	[46]
Diffuse intrinsic pontine glioblastoma	Phase I–II	Veliparib 65 mg/m2 + radiotherapy followed with adjuvant TMZ + veliparib (25 mg/m2)	not applicable	OS: 1 year 29%, 2 year 11%. Well tolerated. No benefit.	[47]
NSCLC Brain metastases	Randomized phase II	Veliparib(50 mg/200 mg/placebo) + WBRT	not applicable	OS: 209d Vs 185d (NS)	[48]

It is worth noting that the ability for some PARP inhibitors to cross the blood–brain barrier has been validated like Veliparib [60]. Promising results are thus expected in clinical studies. A phase I study has found unsafe association of veliparib with radiotherapy and TMZ for glioblastoma because of hematologic complications [45]. Concerning brain metastases, whom radiobiological characteristics are quite different of primary tumors, the association with radiotherapy seems to be safe but results are for the moment disappointing [46, 48].

### PARPi + radiotherapy for digestive system tumors

Like for glioblastoma, radiation therapy plays an important role in the treatment of locally advanced pancreatic cancers, but its effect is limited by the sensitivity of adjacent normal tissues (Duodenum, small bowel…), and by the innate radioresistance of these cancers. Germline mutations in BRCA1/2 define a molecular subgroup of pancreatic cancers which in some populations has a prevalence as high as 17% [61]. According to these arguments, logically strategies of radiosensitization with PARPi have been developped. *In vitro* enhancement ratios vary from 1,2 to 1,5 with conventional radiotherapy until 1,98 with protontherapy [55–59].

Moreover, the effect of radiosensitization was greater for high LET [66].

Authors from Strasbourg laboratory are working on the combination of PARPi (olaparib) and gemcitabine after conventional and high dose photon radiation in order to radiosensitize pancreatic cancer cell lines. The first data have been obtained on three BRCA-wild type pancreatic cancer cell lines (PANC-1, AsPC-1 and MIA PaCa-2). The clonogenic survival responses showed that all cell lines could be radiosensitized with olaparib through an increase of unrepaired DSBs and a block in G2 phase. The radiosensitization with olaparib was higher than with gemcitabine. Furthermore, radiosensitization was higher with high dose per fraction. Finally, in radioresistant cell line PANC-1, olaparib and gemcitabine have a synergistic radiosensitization effect (Waissi et al. [67]). Those data are promising and need further investigations.

*In vivo* results are quite surprising with no increase of growth tumor delay [64, 68], and no differences between BRCA wild type and mutated tumors responses were detected. Nevertheless a phase I study is ongoing and evaluates the association of Veliparib with gemcitabine and radiotherapy for locally advanced pancreatic cancer [69]. First results should be published in 2018. Olaparib is actually investigated alone as maintenance therapy for metastatic pancreatic cancer in phase III studies [70].

Others studies concern mainly colo-rectal cancer with interesting results for *in vivo* studies. Combination of radiotherapy with Veliparib and Irinotecan leads to a threefold increase of growth tumor delay in Shelton *et al*. study [71]. The phase I study LARC, assessing association of Veliparib, Capecitabine and radiotherapy for stage II-III rectal cancer, shows comfortable safety results with promising anti-tumor activity with respectively 72%, 28% and 70% for tumor downstaging, pathologic complete response and sphincter sparing surgery [72]. In conclusion, pre-clinical data show an enhancement ratio median value for pancreatic cancer equal to 1,4 and 1,45 for colo-rectal cancer (Table 3).

**Table 3:** Studies concerning PARPi radiosensitization for digestive system tumors

Digestive tract					
Cell lines / tumor	Phase	PARP Inhibitor	ER	Comments	Ref
Colorectal cancer cell lines (LoVo, SW620)	*In vitro In vivo*	AG14361 0,4 μM AG14361 15 mg/kg/day	Yes (not communicated)	Until additional 18d of delay in tumor growth	[73]
Colorectal cancer cell lines (HCT116)	*In vivo*	Veliparib (25 mg/kg/day)	Not applicable	Median survival time increased from 23 to 36 days	[60]
Colorectal cancer cell lines (HCT116)	*In vitro*	Olaparib (1 μM)	Yes (not communicated)	No radiosensitization of normal intestinal cell line. Increased effect with Combined Chk1 inhibitor	[65]
Colorectal cancer cell line (DLD-1)	*In vitro*	Olaparib (1 μM) +/− Camptothecine	1,20 (alone) to 1,45 (combination)	Increase of G2/M phase cells and reduced S phase cells.	[74]
Colorectal cancer cell lines (HCT116)	*In vitro In vivo*	Veliparib (5 μM) + Irinotecan or oxaliplatine or 5FU	*In vitro*: enhanced ratio 1.60 to 1,82	Until additional 11d of delay in tumor growth	[71]
Colorectal cancer cell lines (HCT116)	*In vitro*	Olaparib and Niraparib (1 μM)	Yes (not communicated)	Reduction of clonogenic survival. Increased autophagy and senescence, but not apoptosis.	[75]
Pancreatic cancer cell lines (Miapaca-2)	*In vitro*	In vitro GPI-21016 (3 μM)	1,4	None	[36]
Pancreatic cancer cell lines (Miapaca-2, MPanc-96)	*In vitro*	Olaparib (1 μM)	1,5	Increased effect with Combined Chk1 inhibitor	[65]
Pancreatic cancer cell lines (Miapaca-2)	*In vitro*	Olaparib (1 μM)	Low LET (γ): 1,4 High LET (Carbon 13keV/um): 1,2 High LET (Carbon 70keV/um) : 1,4	Enhancement ratio reaches 2,5 with 5μM and High LET radiation	[63]
Pancreatic cancer cell lines (MiaPaCa-2, Panc-1,Capan-1, AsPC-1)	*In vitro*	Rucaparib (1 μM) + Gemcitabine	Enhanced ratio: present	Best ratio for BRCA2 mutated Capan-1 cell line.	[62]
Pancreatic cancer cell lines (MiaPaCa2,AsPC-1)	*In Vitro In Vivo*	Olaparib (1 μM) Olaparib (60 mg/kg)	1,2 for MiaPaCa2 1,2 for AsPC-1.	No difference for tumor growth delay.	[64]
Pancreatic cancer cell lines (OCIP 23, OCIP 28)	*In vivo*	Olaparib (150 mg/kg)	Not applicable	No difference for tumor growth delay. No radiosensitization observed in the BRCA2 germline mutant tumor	[68]
Pancreatic cancer cell lines (Miapaca2, PDA)	*In vitro In vivo*.	LT 626 (10μM)	Yes (not communicated)	Synergic effect on Isobolograms	[76]
Pancreatic cancer cell lines (Miapaca-2)	*In vitro*	Olaparib (5 μM) + Protontherapy	1,59 at entrance region 1,98 in the bragg Peak	Increased G2/M arrest	[66]
Hepatocarcinoma cell lines (HepG2, PLC-PRF-5)	*In vitro*	Veliparib (10 μM)	1,48 for HepG2 1,17 for PLC-PRF-5	None	[77]
Locally advanced rectal cancer	Phase Ib	Veliparib + Capecitabine + RT	Not applicable	Tumor downstaging 71% of 31 evaluable pts; Pathologic complete response 29%. Acceptable safety profile.	[78]
Locally advanced pancreatic cancer	Phase I	Veliparib + Gemcitabine+ RT	Not applicable	Safety data	[69]
Advanced solid tumors and carcinomatosis	Phase I	Veliparib + whole abdominal RT	Not applicable	3% objective response. No significant.	[79]

### PARPi + radiotherapy for head and neck cancers

Current treatment regimen consists in a combination of radiotherapy with chemotherapeutics agents such as Cisplatin, 5FU, or Cetuximab and offers great effectiveness but often with unacceptable levels of toxicity [80]. Then we need studies about new radiosensitizers. Güster *et al*., has showed that addition of Olaparib to irradiation, for HPV positive tumors, caused substantial radiosensitization [81]. On the opposite Nickson *et al*., have described the best radiosensitizing effect for HPV negative cell lines with enhancement ratio reaching 3,34 [82]. PARP inhibition could serve as a substitute of Cisplatin, in the context of de-intensified protocols, for HPV positive head and neck tumors, so as to avoid systemic toxicity of conventional chemotherapy [81]. As for other types of tumor, HR defective cells lines are more sensitive to the combination with PARPi [83]. PARPi seem to be able to attenuate the nuclear translocation of EGF-R normally induced by DNA damages. This could lead to lower nuclear interaction between EGFR and DNA PK, and consequently lower DNA repair by c-NHEJ [84, 85]. Median enhancement ratio obtained from pre-clinical data is 1,35 for head and neck tumors. The first phase I study, for locally advanced tumors in heavy smockers, showed promising results but follow-up is for the moment quite short [86] (Table 4).

**Table 4:** Studies concerning PARPi radiosensitization for head and neck tumors

Head and neck tract					
Cell lines/tumor	Phase	PARP Inhibitor	ER	Comments	Ref
HNSCC cell lines (JHU006 and JHU012)	*In vitro In vivo*	GPI-21016 (7 μM)	Yes (not communicated)	Apoptotis increased of 51% for the combinated treatment versus radiotherapy alone. Significant inhibition of tumor growth.	[87]
HNSCC cell lines (UM-SCC1, UM-SCC5, UM-SCC6, Fadu)	*In vitro*	Veliparib (1–10 μM)	Yes (not communicated)	Combination Reduced colony forming ability of cells by 70–95% Vs 30–40% with radiotherapy alone.	[84]
Human HNSCC cell lines (UTSCC5, UTSCC8, UTSCC14, UTSCC15, UTSCC45, FaDu, Cal33, SAS, HSC4 and XF354)	*In vitro*	Olaparib (1 μM)	1,35 to 1,61	1,61 for HR deficient cells 1,35 for HR efficient cells	[83]
HNSCC HPV-p16+ cancer cell lines (93-VU-147T, UM-SCC-47, UTSCC-45,UD-SCC-2, UPCI-SCC-154)	*In vitro*	Olaparib (1 μMz)	Yes (not communicated)	On contrast to cetuximab, the addition of the PARP inhibitor olaparib resulted in a substantial radiosensitization of all HPV(+) cell lines.	[81]
HNSCC cancer cell lines (UT-SCC-12A, UT-SCC-20A,UT-SCC-24B, UT-SCC-30, UT-SCC-45 and UT-SCC-60B)	*In vitro*	Olaparib (1 μM)	1,11 to 1,61	Extent of radiosensitization depends on olaparib dose and BRCA2 status	[88]
Oropharynx HNSCC cancer cell lines (UMSCC6, UMSCC74, UMSCC47, UPCI-SCC090)	*In vitro*	Olaparib (0,1 μM)	1,51 for UMSCC47 3,34 for UMSCC6 1,74 for UMSCC74A	Better results for HPV–cell lines.	[82]
Nasopharyngeal carcinoma cell lines (C666-1, CNE2, HNE1, HONE1)	*In vitro In Vivo*	Olaparib (1 μM) Olaparib (50 mg/kg)	Yes (not communicated)	Strong tumor inhibitory effect in xenograft models. No synergism on nasopharyngeal epithelial cells	[89]
Nasopharyngeal carcinoma cell lines (CNE2)	*In vitro*	3 Amino Benzamide	Yes (not communicated)	Increases the apoptosis rate from 27 for radiation alone to 31% for combination treatment. Significant decrease of proliferation rate	[90]
Heavy smokers with LA-HNSCC	Phase I	Olaparib + Cetuximab + IMRT	Not applicable	Median follow up : 14 months. 3 recurrences. Toxicity acceptable	[86]

### PARPi + radiotherapy for urologic cancers

In the case of urologic cancer, PARPi are today mainly developed for prostate cancer. Relevant studies have identified genomic defects in DNA repair in 20–30% of advanced castration-resistant prostatic cancer cases. A proportion of them are germline aberrations and heritable [91]. This is the first argument for investigating PARP inhibitors or prostate cancer treatments. After publication of high response rate in a phase II study Olaparib has been approved by FDA for the treatment of metastatic castration resistant prostate carcinoma. In this study, 88% of patients with alteration of DNA repair genes (BRCA1&2, ATM, Fanconi anemia genes) had an objective response [91]. The second argument depends on the prevalence of TMPRSS2-ERG gene fusion in prostate cancer which has been reported to range from 40% to 70%, depending on the clinical cohorts investigated [92]. This gene fusion TMPRSS2-ERG interferes with the assembly of c-NHEJ factors at DSBs on the chromatin. By inhibiting c-NHEJ via defective recruitment of XRCC4 and impaired DNA-PKcs phosphorylation, TMPRSS2-ERG rearrangement may reveal a “synthetic lethal” interaction with HR, blocking repair of lesions at collapsed DNA replication forks induced by PARPi [93]. According to the different cell line, radiation enhancement ratio is comprised between 1,05 and 2,2, with much higher effects for TMPRSS2-ERG gene fusion or PTEN deficient cells. It is worth noting that for chronic hypoxia condition, which is a way of radioresistance of prostate cancer, radiosensitizing effect seems better [94]. There is not any clinical trial combining PARPi and conventional radiotherapy for the moment, but a phase Ib with Radium 223 and Niraparib will open soon (NCT03076203). To sum up pre-clinical studies show a median enhancement ratio of 1,3 for prostatic cancer (Table 5).

**Table 5:** Studies concerning PARPi radiosensitization for prostate tumors

Urologic cancer					
Cell lines / tumor	Phase	PARP Inhibitor	ER	Comments	Ref
Prostate carcinoma cell lines (DU145)	*In vitro*	4-amino-1,8-naphthalimide (ANI) (20 μM)	1, 3	None	[95]
Prostate carcinoma cell lines (DU145)	*In vitro*	*In vitro* GPI-21016 (5 μM)	1, 7	None	[36]
Prostate carcinoma cell lines (DU145, 22RV1)	*In vitro*	Veliparib (2, 5 μM)	1, 25 (Under Hypoxia)	SF2 of DU145 and 22RV1 cells decreased from 0.44 and 0.36 to 0.27 and 0.20, respectively.	[96]
Prostate carcinoma cell lines (PC3, DU145)	*In vitro In vivo*	Veliparib (10 μM) Veliparib (25 mg/kg)	Yes (Not communicated)	More Significant decrease of survival fraction for PC3 thant DU-145 *in vitro*. Significant delay in tumor regrowth only for PC3.	[97]
Prostate carcinoma cell lines (PC3, DU145, LNCaP,VCaP)	*In vitro*	Rucaparib (≤ 2, 5 μM)	Yes (Not communicated)	More effective for PTEN deficient cells or with TMPRSS2-ERG gene fusion	[98]
Prostate carcinoma cell lines (PC3, DU145)	*In vitro In vivo*	Olaparib (1 μM) Olaparib (100 mg/kg)	1, 12 to 1, 52	1,12 for ERG-, 1, 52 for Erg+.*In vivo*radioresistance in ERG+ can be overcome through inhibition of PARP1.	[99]
Prostate carcinoma cell lines (LNCaP)	*In vitro*	Niraparib (1 μM)	1, 43	Did not radiosensitize human cells derived from normal tissues	[100]
Prostate carcinoma cell lines (PC3, DU145, LNCaP)	*In vitro*	Olaparib (1 μM)	1, 05 for DU 145 (NS) 1, 3 to 2, 2 for PC3, LNCAP.	In non-responders, the induced DSBs are repaired exclusively by NHEJIn responders, PARP1-EJ shares NHEJ in repairing the DSBs induced after IR	[101]
Prostate carcinoma cell line (22Rv1)	*In vitro In vivo*	Olaparib (1 μM) Olaparib (100 mg/kg)	1, 7	1, 2 under acute Hypoxia, 1, 8 under chronic hypoxia. In vivo : Growth delay increased of 6,06 days (ns)	[94]
Prostate carcinoma cell lines (PC3, LNCaP)	*In vitro*	Rucaparib (2, 5 μM)	Yes (Not communicated)	Most effective for TMPRSS2-ERG gene fusion cells	[93]
Prostate carcinoma cell lines (PC3, VCaP)	*In vitro*	Rucaparib (3 μM), Olaparib (3 μM)	Yes (Not communicated)	SF2 from 0, 50 to 0,12 for olaparib SF2 from 0, 45 to 0, 13 for rucaparib	[102]

### PARPi + radiotherapy for lung cancers

A third of non small cell lung cancers (NSCLC) patients are diagnosed at a locally advanced stage. Radiation therapy instead of surgery is so a standard of care t for these patients. Despite technical advances in radiation therapy, the local tumor control remains suboptimal due to radioresistance. Multiple *in vitro* and *in vivo* studies have been realized, assessing potential role of PARPi as radio sensitizer. Enhancement ratio values are comprised between 1,1 and 1,62 with modern PARP inhibitors under oxia (21%O_2_) conditions for NSCLC. Under hypoxia conditions, which is closer to the clinical reality, enhancement ratio reaches 2,87 suggesting that hypoxia (1%O_2_) induces contextual synthetic lethality with PARP inhibition *in vitro* [103]. Small cell lung cancer (SCLC) cell lines, which show a high expression level of PARP-1 [104], are also radio sensitized by PARPi [105]. Albert *et al*., showed a decrease in *in vitro* endothelial tubule formation with Veliparib/radiation combination treatment, and revealed decreased vessel formation *in vivo*, suggesting that, this strategy may also target tumor angiogenesis [106]. Few phase I clinical trials are actually recruiting patients. In conclusion pre-clinical data show a median enhancement ratio of 1,5 for lung cancer (Table 6).

**Table 6:** Studies concerning PARPi radiosensitization for lung tumors

Lung cancer					
Cell lines / tumor	Phase	PARP Inhibitor	ER	Comments	Ref
NSCLC cell lines	*In vitro*	3 amino benzamide (8 mM)	No	None	[107]
NSCLC cell lines (HX147c)	*In vitro*	3 amino benzamide (8 mM)	No	Absence of radiosensitization whatever the dose rate.	[44]
NSCLC cell lines (H460)	*In vitro In vivo*	Veliparib (5 μM)	1,27	Tumor growth delay increased of 6 days.	[106]
NSCLC cell lines (Calu-6,A549)	*In vitro In vivo*	Olaparib (1 μM)	1,3 to 1,5	Tumor growth delay increased of 10 Days	[108]
NSCLC cell lines (H1299)	*In vitro*	Veliparib (2, 5 μM)	1,38	Same results under hypoxia (SER = 1,38)	[96]
NSCLC cell lines (H1299)	*In vitro*	Olaparib (1 μM)	1,1 to 1,2	Radiosensitization regardless p53 status	[74]
NSCLC cell lines (H1299, A549)	*In vitro*	Olaparib (1 μM)	1,3 to 2,2	No effect on A549. Hypothesis: In non-responders, the induced DSBs are repaired exclusively by NHEJIn responders, PARP1-EJ shares NHEJ in repairing the DSBs induced after IR	[101]
NSCLC cell lines (Calu-6,A549,H1299,H460)	*In vitro*	Niraparib (1 μM)	A 549: 1,32 H1299 : 1,34 H460: 1,42 Calu6: 1,61	Radiosensitizing effect of niraparib is independent of p53-status.	[100]
NSCLC cell lines (H460)	*In vitro*	Olaparib (1μM)	Yes (not communicated)	p53 defective cells are more radiosensisitized by olaparib.	[65]
NSCLC cell lines (H460,A549,Calu-6)	*In vivo*	Niraparib(1 μM)	Yes (*In vivo*)	*In vivo*: 1,4-2,2 (growth delay). 1or 2 Fraction/day was found to be highly and similarly effective	[109]
NSCLC cell lines (Calu-6,Calu-3)	*In vitro In vivo*	Olaparib (5 μM)	Calu 3: 2,18 Calu 6: 1,95	2,87 under hypoxia 2,44 under hypoxia Tumor growth delay increased only for Calu 6.	[103]
NSCLC cell lines (A549)	*In vitro*	Olaparib (5 μM) + Protontherapy	1,45 at entrance region 1,89 in the bragg Peak	Increase of G2/M arrest	[63]
NSCLC cell lines (H1299, H460)	*In vitro In vivo*	LT 626 (10 μM) LT 626 (20 mg/kg)	Yes (not communicated)	Survival was increased from 13 to 20 d. Tumor burden was significantly lower.	[76]
SCLC cell lines (H146, DMS153)	*In vitro*	Veliparib (5 μM)	Yes (not communicated)	≈30% decrease of survival fraction at 2 Gy.	[105]

### PARPi + radiotherapy for breast and gynaecologic cancers

With ovary cancer, breast cancer is the most known tumor to get BRCA1 or 2 mutations, and is thus a candidate for PARPi radiosensitization. Nevertheless, surprisingly, PARP1 inhibition improves the therapeutic index of radiotherapy independent of breast cancer subtype or BRCA1 mutational status. Among breast cancer subtypes, HER2 and luminal cancer cells seem to get better enhancement ratio [110]. While it remains dogma that IR and genotoxic agents mediate their lethal effects via enhanced apoptosis, necrosis or mitotic catastrophe, Efimova *et al*. showed that for Breast cancer cell lines PARP inhibitors may have a significant impact by inducing senescence [111]. Median value of enhancement ratio induced by PARPi for breast cancer is 1,36. The safety datas of phase I study of Jagsi *et al*. about loco regional radiotherapy for local recurrence of Triple negative breast cancer are comforting, with one unexpected grade IV toxicity on 30 patients [112] (Table 7).

**Table 7:** Studies concerning PARPi radiosensitization for breast and gynecologic tumors

Gynaecologic and breast cancer					
Cell lines / tumor	Phase	PARP Inhibitor	ER	Comments	Ref
Breast Cancer cell line (MCF7)	*In vitro In vivo*	Veliparib (10 uM, 0,5 mg Bid)	Yes (Not communicated)	Colony formation following 2 Gy decreased from 29.7 to 11.3% with veliparib. The antiproliferative effects of 3 Gy enhance from 41 to 27%	[111]
Breast Cancer cell line (MDA-MB-231)	*In vivo*	Niraparib (25 to 50 mg/kg)	Yes (*In vivo*)	*In vivo*: increase growth delay x 1,9	[109]
Breast Cancer cell lines (MDA-MB-231, MDA-MB-436)	*In vitro In vivo*	Niraparib (1 μM)	1, 25 to 1,36	Normal breast cell line MCF 10A only slightly radiosensitized.	[100]
Breast Cancer cell line (ZR75-1, MDA-MB-231, MDA-MB-468, MDA-MB-453, HCC 1954, HCC 1937)	*In vitro In vivo*	Veliparib (2,5 uM) Veliparib 100 mg/Kg	1,22 to 2,31	*In vivo* : Tumor volume doubling time ≈ x2	[110]
Triple negative breast cancer	Phase I	Veliparib + 50 Gy	Not applicable	30 patients. Safety Data	[112]
Cervix carcinoma cell line (Hela)	*In vitro*	Olaparib (1 μM)	1,3 to 2,2	PARP inhibition significantly increased the number ofpersistent gH2AX foci at 24h but not at 1h	[101]
Cervix carcinoma cell line (HX 155, 156, 160)	*In vivo*	3-Amino-benzamide (8 mM)	1,02 to 1,37	None	[113]
Cervix carcinoma cell line (Hela)	*In vitro*	4-amino-1,8-naphthalimide (30 μM)	No	No significant increase of radiosensitivity	[15]
Cervix carcinoma cell line (HX 156c)	*In vitro*	3-Amino-benzamide (8 mM)	1,16 to 1,18	Absence of radiosensitization whatever the dose rate.	[44]

### PARPi + radiotherapy for rare cancers

**Figure 3:**
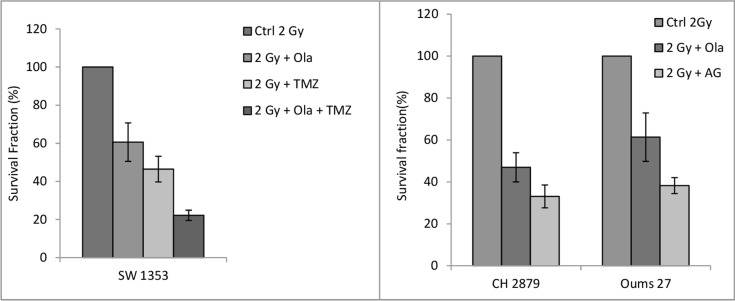
Clonogenic survival rate of chondrosarcoma cell after protontherapy sensitization with alkylant agents and PARPi Chondrosarcoma cells were first cultured with Temozolomide and /or PARPi for 2 hours, then irradiated with proton beam at 2 Gy (62 MeV.u^−1^, SOBP, 1,1 keV u^−1^ at LNS, Catania, Italy) and then left overnight at 37°C. Cells were then seeded at low density for subsequent clonogenic assay, as previously described [116]. Left: survival fraction of SW1353 chondrosarcoma cells after 2 Gy proton alone, and with olaparib (2 μM), temozolomide (Sigma-Aldrich ref T2577) (20 μM) and olaparib with temozolomide (2 μM + 20 μM respectively). Right: survival fraction as a function of different chondrosarcoma cell lines. CH 2879 and Oums 27 are two other chondrosarcoma cell lines, showing that the variability of the response to the treatment is related to the cell line used. Cells were irradiated alone or with Olaparib (Focus biomolecules Ref 102154) at 2 μM, or AG (AG14361, Tebu-Bio, ref 27602-3) at 0.4 μM. Each treatment is estimated as a percentage of the control sample, repeated 3 times.

Few authors expressed an interest in rare tumors such as Ewing sarcoma and have obtained promising pre-clinical results, but data stay poor [107, 114]. It is also the case for chondrosarcoma which is however considered as one of the most radioresistant tumor. Our laboratory is working on combination of PARPi (AG15361, Olaparib, and Talazoparib) and conventional radiotherapy, protontherapy or hadrontherapy in order to radiosensitize chondrosarcoma cells. First data with conventional radiotherapy are very interesting and deserve further investigations. All clonogenic survival data with olaparib showed radiosensitization of Oums 27 or CH 2879 cell lines (PTCOG 2016, Chevalier *et al*. [115]). There are still on going investigations (Figure 3).

## DISCUSSION

Radiation therapy plays a central role in cancer therapeutics, as an adjuvant therapy and above all as first treatment, alone or with radiosensitizer, for low-operable tumors such as glioblastoma or advanced lung cancers. Nevertheless, cure rates stay disappointing, and concomitant chemotherapy generate important systemic toxicity, requiring search for new radiosensitizers. Ideal radiosensitizing agents should have two main qualities: on the first hand to offer better protection to surround healthy tissues, on the other hand, to increase anti tumoral efficiency, with hope to improve therapeutic ratio. Since the last ten years PARP seems to be one of the most interesting protein to target, in association with radiotherapy because particularly of its role in DNA repair. We have reported more than fifty *in vitro* or *in vivo* studies which have investigated PARPi as radiosensitizer with attractive enhancement ratio comprised between 1,04 and 2,87 for *in vitro* data (Tables 2, 3, 4, 5, 6, 7). PARPi could even bypass some radioresistant mechanisms such as hypoxia [96, 103]. Nevertheless, *in vivo* results are often less promising, and only 9 phase I-II studies get published results, sometimes negative. Nine other phase I-II clinical trials (clinicaltrials.gov) are actually recruiting, all using Olaparib or Veliparib in combination with radiotherapy or radio-chemotherapy for soft tissue sarcoma, breast cancer, lung cancer, rectal cancer or esophageal cancer. During the 2 or 3 next years, they should bring some new information concerning the potential of PARPi as radiosensitizer.

It is important to note that, combination treatment appears to promote a state of growth arrest instead of cell death, it is legitimate to speculate that radiosensitization could be a consequence of an increase in the extent of senescence [75, 97, 111]. The next critical question is whether the growth arrest is sustained and irreversible or transient and reversible, and consequently if this combination approach is so useful for improving long-term endpoints.

A current interrogation remains that radiosensitizing effect on high proliferating non tumoral tissues, like mucosa or bone marrow, is quite unknown. Effectively *in vitro* studies including investigations with normal cells lines are very limited [63, 117] and there are too few of phase I studies to bring some conclusions. Nevertheless as single agent, in phase III study, Olaparib increased the risk of myelodysplasia or acute myeloid leukemia [118]. These secondary effects could be enhanced if large field spine or pelvic radiotherapy was associated. Waiting for more data, and to avoid unexpected late toxicity, we should pay attention to the use of hadrontherapy, stereotactic radiotherapy or radionuclides in combination with PARPi. These techniques offer better ballistic and superior biological efficiency, bringing a promise of improvement of the therapeutic ratio [119].

If BRCA and PTEN mutations are actually known for being predictive of high efficiency of PARP inhibitor [120], accurate biomarkers for response and resistance to PARPi are still needed. Intratumoral levels and activity of the PARP does not correlate with clinical responses in patients and shouldn't be considered as predictive of response [121]. On the contrary, homologous recombination assay, targeted multiplex sequencing, mutational signatures and alterations in genome structure, functional assay, are potential solutions to identify biomarkers [122]. For example Olaparib as single agent for patients suffering from prostate cancer with genetic aberrations in DNA-repair genes has showed significantly higher efficacy with 88% overall response rate. In this study, 12 genes were evaluated: BRCA1, BRCA2, ATM, FANCA, CHK2, PALB2, HDAC2, RAD 51, MLH3, ERCC3, MRE 11 and NBN [91]. Future phase I or II studies should systematically include tumoral DNA Repair capacity status, in order to better select patient who will benefit from the treatment.

Finally we come to the conclusion that the permanent development of new targeted therapies makes radiotherapist hope for new interesting pair with radiotherapy. Combinations of PARPi with radiotherapy seem very promising but clinical data are still lacking. Radiotherapists and searchers should pay attention to include ancillary studies, in addition to clinic ones, in order to develop biomarkers. The next ten years will be crucial for judging the real potential of PARPi as radiosensistizer.
